# Potassium current inactivation as a novel pathomechanism for *KCNQ2* developmental and epileptic encephalopathy

**DOI:** 10.1111/epi.18427

**Published:** 2025-04-28

**Authors:** Ingride Luzio Gaspar, Gaetano Terrone, Giusy Carleo, Lidia Carotenuto, Francesco Miceli, Gabriella De Vita, Maurizio Taglialatela

**Affiliations:** ^1^ Section of Pharmacology, Department of Neuroscience University of Naples Federico II Naples Italy; ^2^ International School of Advanced Studies University of Camerino Camerino Italy; ^3^ Section of Child Neuropsychiatry, Department of Translational Medical Sciences University of Naples Federico II Naples Italy; ^4^ Section of Medical Genetics, Department of Molecular Medicine and Medical Biotechnology University of Naples Federico II Naples Italy; ^5^ CEINGE Advanced Biotechnology Naples Italy

**Keywords:** developmental encephalopathies, epilepsy, potassium current inactivation, *KCNQ2*, loss‐of‐function variant, potassium channels

## Abstract

De novo variants in *KCNQ2* cause neonatal onset developmental and epileptic encephalopathy (*KCNQ2*‐DEE; Online Mendelian Inheritance in Man #613720), most often by loss‐of‐function in vitro effects. In this study, we describe a neonatal onset DEE proband carrying a recurrent de novo *KCNQ2* variant (c.794C>T; p.A265V) affecting the pore domain of *KCNQ2*‐encoded Kv7.2 subunits. Whole‐cell patch‐clamp measurement in a mammalian heterologous expression system revealed that, when compared to wild‐type Kv7.2 channels, channels containing Kv7.2 A265V subunits displayed (1) reduced maximal current density; (2) decreased voltage‐dependence of activation; and (3) an unusual inactivation process, with a 50% current reduction during 1–2‐s depolarizing pulses at voltages > 0 mV. These effects were proportional to the number of mutant subunits incorporated in heteromeric channels, being overall less dramatic upon coexpression with Kv7.2 or Kv7.2 + Kv7.3 subunits. These results reveal current inactivation as a novel pathogenetic mechanism for *KCNQ2*‐DEE caused by a recurrent variant affecting a critical pore residue, further highlighting the importance of in vitro functional assessment for a better understanding of disease molecular pathophysiology.

## 1 INTRODUCTION

The *KCNQ2* gene encodes for Kv7.2 voltage‐gated potassium (K^+^) channel subunits contributing to the M‐current (I_KM_), a subthreshold, slowly activating, and noninactivating current that reduces neuronal excitability and causes spike frequency adaptation.^1^, ^2^ Heterozygous *KCNQ2* pathogenic variants cause a broad phenotypic spectrum of mostly neonatal onset epilepsies; these range from self‐limited familial neonatal epilepsy (SLFNE) to severe sporadic cases of developmental and epileptic encephalopathy (*KCNQ2*‐DEE) most often caused by de novo missense variants. When studied in vitro, most disease‐causing variants reduce Kv7.2 function, prompting loss‐of‐function (LoF) effects.^3^ A correlation exists between the extent of in vitro functional impairment and the clinical disease severity; SLFNE‐causing variants mostly induce milder LoF effects when compared to DEE‐causing pathogenic variants.^3^ The molecular mechanisms responsible for the decrease in I_KM_ function by disease‐causing *KCNQ2* variants are heterogeneous and include changes in subunit expression levels caused by transcriptional and translational impairment, as well as dysfunction(s) in ion permeation, selectivity, gating, and regulation by critical endogenous proteins or cofactors (phosphatidylinositol 4,5‐bisphosphate in particular).^2^, ^4^ Most notably, distinct molecular pathomechanism(s) are triggered by different disease‐causing variants; therefore, functional analysis of novel and recurrent *KCNQ2* mutations is crucial for a better understanding of the disease pathophysiology and to identify the most appropriate pharmacological intervention. We herein describe a DEE proband carrying a recurrent de novo *KCNQ2* variant (A265V) affecting the Kv7.2 pore domain and reveal current inactivation as a novel pathogenetic LoF mechanism for the disease.

## 2 MATERIALS AND METHODS

### 2.1 DNA extraction and genetic screening

Written informed consent for genetic analyses was obtained from the patient and her parents. Genomic DNA was extracted from peripheral‐blood lymphocytes using standard procedures. For next generation sequencing (NGS) analyses, a Customized Clinical Panel (Agilent) targeting 5228 genes and covering approximately 17 Mb was used. An in silico panel of 400 genes related to epilepsy and DEE (list available upon request) was filtered for bioinformatic analysis. For each gene, the coding regions, 25 bp in each of the intronic boundaries, and the promoters were analyzed. The average coverage at 50× was 95%. NGS data were filtered using a software pipeline developed in‐house, based on the human reference genome version hg19/GRCH37. Variants were classified following currently available guidelines.^5^ Variant validation and parental segregation were performed by standard Sanger sequencing.

### 2.2 Mutagenesis and heterologous expression

The c.794C>T (p.A265V) mutation was engineered in human *KCNQ2* cDNA cloned in pcDNA3.1 by quick‐change mutagenesis.^6^ Chinese hamster ovary (CHO) cells were transiently transfected using Lipofectamine 2000 with various cDNA amounts and ratios of *KCNQ2*, *KCNQ2* A265V, and *KCNQ3*, according to the experimental protocol. Total cDNA was kept at 3 μg in the transfection mixture. A plasmid encoding for the Enhanced Green Fluorescent Protein (pEGFP; Clontech) was used to identify transfected cells.

### 2.3 Whole‐cell electrophysiology

CHO whole‐cell currents were recorded with the patch‐clamp technique using previously described voltage protocols, solutions, and analysis methods.^6^, ^7^ To measure activation and inactivation kinetics, as well as voltage dependence of activation and inactivation, currents were elicited with 1.5‐s depolarizing steps ranging from −80 mV to +20/+30 mV in 10‐mV increments, followed by an isopotential step at 0 mV.^6^ Conductance values were obtained from *G* = *I*/(*V* − *V*_rev_), where *I* is the peak current during the test depolarization (*V*), and *V*_rev_ is the potassium reversal potential. Data were normalized to maximum peak conductance (*G*_max_) and fit to the following form of a two‐state Boltzmann distribution: *G*/*G*_max_ = (1 + exp (*V*_1/2_ − *V*)/*k*), where *V*_1/2_ is the half‐activation potential, *V* is the test potential, and *k* is the slope factor. Activation kinetics were measured by fitting the activation current trace at 0 mV from the beginning of the depolarizing step until the peak, using a single exponential function to obtain *τ*_act_ values.^7^, ^8^ Inactivation kinetics were measured by fitting the current trace from the peak until the end using a single exponential function, thus obtaining *τ*_inact_ values.^7^ Finally, the extent of inactivation was calculated by dividing current values at the end of the depolarizing step by the peak values.^8^, ^9^ The recovery from the inactivation of Kv7.2 A265V currents was determined using a double‐pulse protocol in which a first pulse to +20 mV (*I*_1_) was followed, after 100 ms (with 250‐ms increment intervals), by a second depolarizing pulse to the same potential (*I*_2_). The interpulse voltages were: −60 mV, −80 mV, and −100 mV. The extent of recovery from inactivation was calculated from the *I*_2_/*I*_1_ ratio as a function of time and voltage; these data were then fitted with a single exponential function to obtain *τ*_rec_.^9^

### 2.4 Statistics

Data are expressed as mean ± SEM. When comparing two independent groups, the unpaired *t*‐test was applied. For multiple groups comparison, a mixed‐effects model was used for repeated measures followed by Sidak multiple comparison test. The robust regression outlier test (*Q* = 10) was performed to identify possible outliers. Statistical significance was accepted if *p* < .05.

## 3 RESULTS

### 3.1 Clinicogenetic features of the proband

**FIGURE 1:**
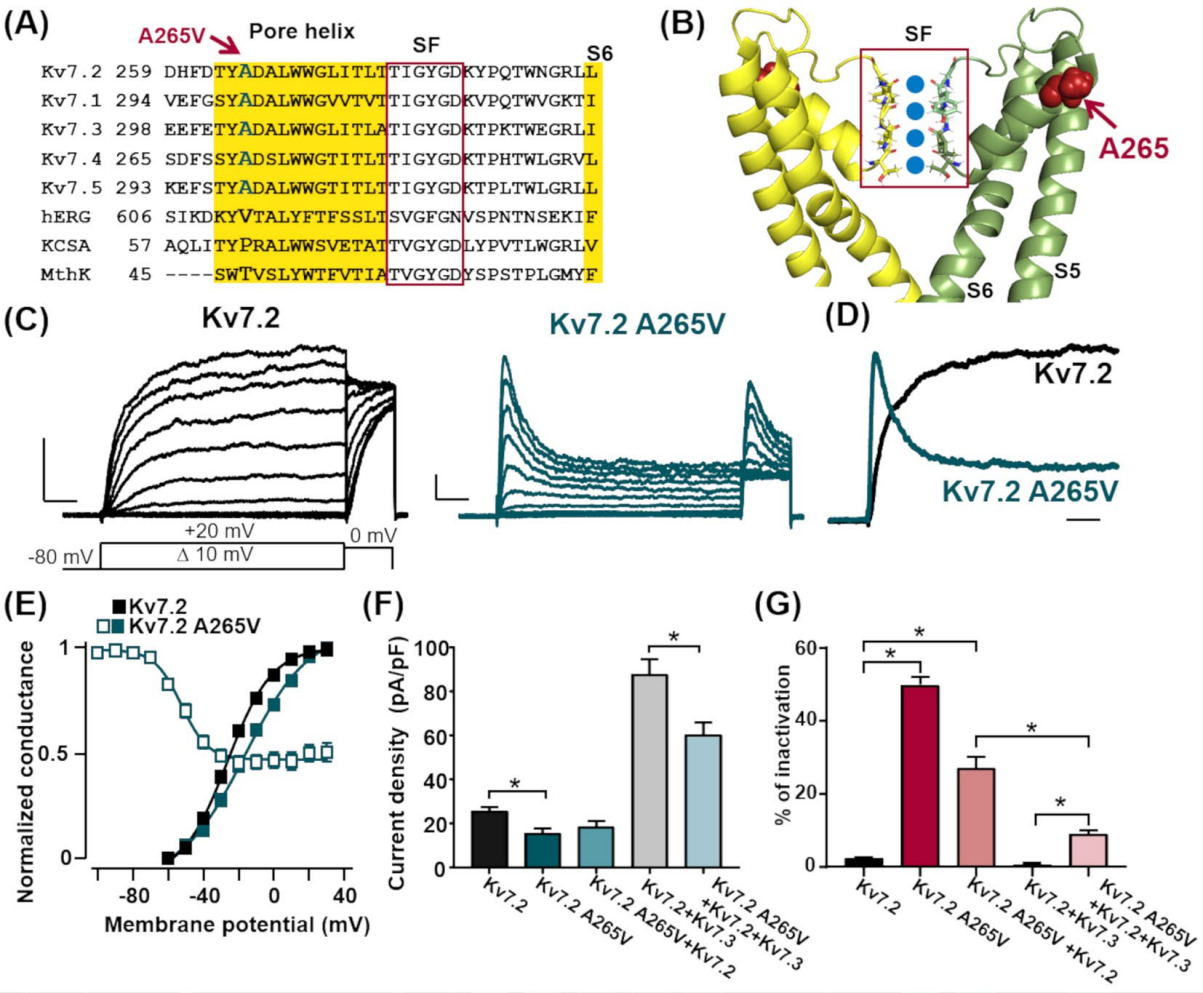
Localization and functional characterization of the Kv7.2 A265V variant. (A) Alignment of the amino acid sequence within the S_5_–S_6_ region of the indicated channels. The position of the A265 residue in  a Kv7.2 subunit is indicated by an arrow. The regions in yellow correspond to the pore helix and the beginning of S6. The boxed area corresponds to the selectivity filter (SF). (B) Kv7.2 structure (Protein Data Bank code: 8J05) of S_5_, S_6_, and the intervening linker, including the pore domain from two opposite subunits; the other two subunits present in functional tetrameric channels have been removed for clarity. The A265 side‐chain atoms are shown as spheres. (C) Macroscopic currents from Kv7.2 and Kv7.2 A265V channels, in response to the indicated voltage protocol (scale: 200 pA, 200 ms). (D) Superimposed and normalized current traces of Kv7.2 (black) and Kv7.2 A265V (dark green) channels at +20 mV (time scale: 200 ms). (E) G/V curves for the indicated channels; conductance (G) values at each potential (V) were calculated by dividing the peak current values by the K^+^ equilibrium potential. (F) Maximal current density at 0 mV recorded from cells transfected with the indicated cDNAs. (G) Percent of inactivation at +20 mV in the current carried by the indicated channels (same as panel E). *p < .05 versus respective control.

The proband (Patient 1 in Table 1) is currently a 21‐month‐old female, born at 37 weeks of gestation from healthy Caucasian nonconsanguineous parents. Starting from the second day of life, she exhibited multiple daily focal motor seizures resistant to phenobarbital, levetiracetam, and pyridoxine but responsive to carbamazepine. The electroencephalogram (EEG) pattern was discontinuous, with a burst of theta activity intermixed by sharp waves, especially over the centrotemporal leads of both hemispheres, separated by asymmetrical periods (3–7 s) of marked generalized voltage attenuation. At 18 months of life, an assessment by Griffiths Mental Developmental Scales‐III showed a developmental quotient of 50 (<1^st^ percentile), with an equivalent age of 10 months, suggestive of a global developmental delay. A sleep EEG showed epileptic abnormalities over the right frontocentrotemporal leads spreading to the anterior regions of the contralateral hemisphere. At present, the patient is seizure‐free on a drug‐combined regimen of carbamazepine (36 mg/kg/day) and phenobarbital (1.4 mg/kg/day), as detailed in Table 1. Trio genetic testing revealed a heterozygous de novo *KCNQ2* missense variant (c.794C>T; p.A265V), affecting a residue in the extracellular portion of the S_5_–S_6_ linker of the Kv7.2 subunit. This residue is highly conserved among Kv7 subunits, but not in other channel families (Figure 1A). The Kv7.2 A265V variant is highly recurrent,^11^, ^12^, ^13^, ^14^, ^15^, ^16^ but no in vitro functional assessment has ever been carried out; thus, electrophysiological assays were deemed necessary.

**TABLE 1:** Clinical features of patients carrying the *KCNQ2* c.794C>T (p.A265V) de novo variant.

Characteristic	Patient					
	1	2	3	4	5	6
Sex	F	M	M	M	M	M
Diagnosis	EOEE (EIDEE)	Ohtahara syndrome	EOEE (EIDEE)	Ohtahara syndrome	Ohtahara syndrome	EOEE (EIDEE)
SZ onset	2nd day	1st day	2nd day	2nd day	1st day	1st day
Initial symptoms	Generalized hypertonia	Right opsoclonuslike movement, apneic spell	Facial flushing, eye fixation	No cry, poor suck, stiffening and arching with eye rolling		
SZ type	SZ with hypertonic asymmetric posture	Tonic SZ	Tonic SZ	Left‐sided SZ	Temporal‐originated focal tonic SZ	Focal tonic SZ with bilateral symmetrical epileptic spams
SZ frequency	Multiple episodes/day	Unknown	Multiple episodes/day	Unknown	Multiple episodes/day	
SZ outcome	SZ‐free at 1 month	Unknown	SZ‐free at 16 months with CBZ	Died at 3 months	SZ‐free at 2 months	SZ‐free at 6 months
Drug treatment	B6, LEV, PB, CBZ; current: CBZ, PB	B6, CBZ, CZP, VPA, ZNS	CBZ, DZP, MDL, PB, PLP, VPA	PB, PLP, CLB, MDL, VGB	PB, LEV, ACTH, TPM, OXC	PB, OXC, TPM; current: TPM
EEG	Multifocal epileptic abnormalities	Burst suppression	Multifocal sharp waves in the beginning; focal spikes, moving loci	Burst suppression, rolandic spike‐waves, normal background activity	Hypsarrhythmia, burst suppression	Burst suppression
Development	Global developmental delay	Delayed, no eye pursuit	Delayed, mild MR	Unknown	Delayed	Normal
Features	Convergent strabismus, tongue dyskinesias, mild rigidity of upper limbs, truncal hypotonia, fleeting episodes of nystagmus	Myoclonus at the bilateral upper extremities	Spastic diplegia with dyskinesia, athetotic/dystonic movements	Hypotonia, jerking of left arm, stiffening and arching	Premature atrial and ventricular contractions, episodes of tachycardia after an epileptic episode	
Reference	This work	11 (Patient 1754)	12 (Patient 14)	12 (Patient 110)	13	15 (Patient 11)

*Note*: According to recent International League Against Epilepsy classification,^10^ the term EIDEE includes neonates and infants previously classified as Ohtahara syndrome and early myoclonic encephalopathy.

Abbreviations: ACTH, adrenocorticotropic hormone; B6, vitamin B6; CBZ, carbamazepine; CLB, clobazam; CZP, clonazepam; DZP, diazepam; EEG, electroencephalogram; EIDEE, early infantile developmental and epileptic encephalopathy; EOEE, early onset epileptic encephalopathy; F, female; LEV, levetiracetam; MDL, midazolam; MR, mental retardation; OXC, oxcarbazepine; PB, phenobarbital; PLP, pyridoxal phosphate; SZ, seizure; TPM, topiramate; VGB, vigabatrin; VPA, valproic acid; ZNS, zonisamide.

### 3.2 Functional analysis: Homomeric channels

Currents carried by heterologously expressed Kv7.2 channels, similarly to native I_KM_,^17^ display a rather negative activation threshold (~−50 mV), slow activation and deactivation kinetics, and lack of inactivation.^2^, ^6^ When compared to Kv7.2, currents from Kv7.2 A265V homomeric channels activated around similar threshold voltages, but displayed faster activation kinetics (*τ*_act_), reduced maximal current density, a rightward shift in activation gating, and an unusual decrease during the depolarizing pulse, reaching a steady‐state value at approximately 50% of the peak (Figure 1C–G, Table S1); this latter phenomenon is consistent with open‐state inactivation, namely, a decreased stability of the open state, which relaxes toward a nonconductive inactivated state during prolonged activating stimuli. Analysis of the voltage‐dependence of the occupancy of this inactivated state revealed a midpoint potential (*V*_½_) of −54 ± 2 mV and a slope value (*k*) of 8 ± 1 (*n* = 13). As for most inactivating K^+^ currents,^8^ the extent and kinetics of the recovery from the inactivated state in Kv7.2 A265V currents were also voltage‐dependent, being faster and more complete at hyperpolarized potentials (−100 mV) and slower and less complete at more depolarized membrane potentials (−60 mV; Table S1, Figure S1A,B). Homomeric Kv7.2 channels are highly sensitive to blockade by extracellular application of tetraethylammonium (TEA), a nondehydratable analogue of K^+^.^18^, ^19^ A slight decrease in blocking efficacy by .3 mmol·L^−1^ TEA was observed in mutant channels (the percent of blockade was 52% ± 3% vs. 68% ± 3% for Kv7.2 A265V and Kv7.2 channels, respectively; *p* < .05), possibly because of their smaller current amplitude. However, no change was observed in Kv7.2 A265V current inactivation kinetics before and after TEA exposure; the inactivation time constant (*τ*_inact_) was 239 ± 21 ms and 224 ± 35 ms before and after TEA exposure, respectively (*p* > .05; Table S1). Similarly, no change in Kv7.2 A265V current inactivation kinetics was observed when the extracellular K^+^ concentration was raised from 5 to 15 mmol·L^−1^ (*τ*_inact_ = 291 ± 29 ms, *p* > .05 vs. controls; Table S1).

### 3.3 Functional analysis: Heteromeric channels

The A265V mutation found in Patient 1 as well as in all previously described cases (Patients 2–6; Table 1) occurred in heterozygosity. Moreover, native I_KM_ in adult neurons is mainly formed by heteromeric assembly of Kv7.2 and Kv7.3 subunits, the latter encoded by the *KCNQ3* gene.^17^ Therefore, the functional consequences of the A265V variant when mutant subunits were expressed in heteromeric channels together with Kv7.2 (1:1 cDNA ratio) and/or Kv7.2 and Kv7.3 subunits (.5:.5:1 cDNA ratio) were also investigated. When compared to Kv7.2, currents from CHO cells coexpressing Kv7.2 A265V and Kv7.2 subunits were slightly but not significantly decreased in their maximal size (Figure 1F), although their activation was shifted toward more positive potentials (Table S1). In addition, when compared to that of homomeric Kv7.2 A265V channels, the extent of current inactivation upon coexpression of Kv7.2 A265V + Kv7.2 subunits was reduced to almost half (Figure 1G), and the currents showed slower inactivation kinetics (Table S1). When compared to Kv7.2 + Kv7.3 heteromers, currents recorded from cells coexpressing Kv7.2 A265V + Kv7.2 + Kv7.3 subunits showed (1) a decreased maximal size (Figure 1F), (2) a right‐shifted V_½_ (Table S1; Figure S1C,D), (3) a relative size of the inactivating component smaller than that observed in homomeric Kv7.2 A265V‐ or Kv7.2 + Kv7.2 A265V‐expressing cells (Figure 1G), and (4) a non‐statistically significant trend toward faster activation rates (Table S1).

## 4 DISCUSSION

The herein described proband (Patient 1 in Table 1) showed drug‐resistant daily seizures starting in the first days of life, discontinuous EEG, and neurodevelopmental delay, all phenotypic characteristics suggestive of *KCNQ2*‐DEE, an etiology‐specific syndrome recently classified by the International League Against Epilepsy.^10^ Genetic investigation revealed a heterozygous de novo variant (A265V) in the *KCNQ2* gene, confirming the diagnosis. The Kv7.2 A265V variant is highly recurrent; it has been previously found in five patients, all showing phenotypic features consistent with *KCNQ2*‐DEE (Table 1),^11^, ^12^, ^13^, ^15^ as well as in three *KCNQ2*‐DEE individuals for whom no detailed phenotypic information is available.^14^, ^16^ Moreover, one additional *KCNQ2*‐DEE patient (Patient 4 in Weckhuysen et al.^20^) carried the A265P substitution, suggesting that A265 is a mutational hotspot for *KCNQ2*‐DEE.

Our functional analysis revealed that the A265V variant prompts remarkable LoF effects in Kv7.2 channels, confirming that most *KCNQ2*‐DEE variants markedly decrease channel function.^3^ When compared to Kv7.2 channels, currents from homomeric Kv7.2 A265V channels display a reduced maximal density, right‐shifted activation gating (requiring more depolarized voltages to open), and a unique inactivated state. Although these functional effects are clearly consistent with an LoF in vitro phenotype, current carried by Kv7.2 A265V channels also display faster activation rates, a result consistent with a gain‐of‐function (GoF) effect.

Notably, the extent of current inactivation prompted by the Kv7.2 A265V variant is proportional to the average number of mutant subunits present in tetrameric channels, namely four for Kv7.2 A265V homomers, two for Kv7.2 A265V + Kv7.2 heteromers, and one for Kv7.2 A265V + Kv7.2 + Kv7.3 heteromers. This phenomenon is likely to have relevant pathophysiological impact for I_KM_, the molecular composition of which is dynamic and flexible, with most adult neurons expressing Kv7.2 + Kv7.3 heteromers, whereas others express Kv7.2 and Kv7.3 homomers as a function of developmental stage, brain area, cell type, subcellular region, and disease state.^21^, ^22^

Several inactivation mechanisms occur in voltage‐dependent K^+^ channels.^23^ For instance, *Shaker*‐type Kv1 channels display a fast inactivation process (*τ*_inact_ of a few milliseconds) in which a cytoplasmic domain of either the channel or an accessory subunit plugs the open pore to interrupt ion flux via a “ball‐and‐chain” mechanism; given the location of the inactivation particle at the N‐terminal end of the channel, such inactivation mechanism is commonly referred to as “N‐type inactivation.”^24^ Notably, no inactivating particle is present in Kv7 channels, thus the noninactivating characteristics of I_KM_. In other K^+^ channels, or upon removal of the inactivating particle in N‐type inactivating channels, slower “P‐type” or “C‐type” inactivation processes (*τ*_inact_ from hundreds of milliseconds to seconds) were revealed^25^, ^26^; during such slower transitions, ion flow is restricted by conformational changes of the selectivity filter located near the extracellular side of the pore,^27^ as recently confirmed by structural (cryoelectron microscopic) data.^28^ A hallmark of C‐type inactivation is the ability of extracellular cations, such as K^+^ ions or TEA, to compete with C‐type inactivation.^27^ Notably, the A265 residue is positioned away (18.8 Å) from the Kv7.2 selectivity filter (Figure 1B), and application of .3 mmol·L^−1^ TEA_e_ or increasing the extracellular K^+^ concentration from 5 to 15 mmol·L^−1^ did not modify the inactivation rate; both these observations suggest that the A265V substitution triggers a different, noncanonical (non‐N‐type, non‐C‐type) inactivation mechanism. Further investigation is needed to better delineate the atomistic basis for the described functional features.

In conclusion, the recurrent A265V variant in Kv7.2 is associated with a severe phenotype of *KCNQ2*‐DEE and, when expressed in vitro, shows functional characteristics of a “mixed” LoF/GoF, with unusual inactivation features never described before. This observation emphasizes the importance of the functional analysis of novel and recurrent *KCNQ2* mutations and expands the repertoire of mutation‐induced functional changes causing *KCNQ2*‐DEE. Kinetics and extent of inactivation of ion channels are major determinants of their specific physiological functions^27^; the noninactivating properties of I_KM_ are essential for the dampening role exerted by this conductance on neuronal excitability and firing frequency. Therefore, current inactivation prompted by the Kv7.2 A265V variant likely reduces spike‐frequency adaptation, leading therefore to an overall enhancement of neuronal excitability; on the other hand, the faster current activation kinetics conferred by the A265V variant, by reducing/delaying action potential firing and facilitating action potential repolarization, would decrease neuronal excitability. Thus, further work is required to investigate the functional consequences of this variant in distinct neuronal subpopulations.

## FUNDING INFORMATION

This work was supported by the Italian Ministry for University and Research with PRIN2022 (project 2022M3KJ4N to M.T.), and PRIN2022PNRR (project P2022FJXY5 to F.M.; project P2022ZANRF to M.T.); the European Union—Next Generation EU, Mission 4, Component 2, CUP E63C22002170007 (project “A Multiscale Integrated Approach to the Study of the Nervous System in Health and Disease,” MNESYS; to M.T.); the Italian Ministry of Health with Ricerca Finalizzata projects PNRR‐MR1‐2022‐12376528 to M.T. and PNRR‐MCNT2‐2023‐12377937 to M.T.; and the European Joint Program EP Permed 2024 (BeatKCNQ to M.T.). The work was also supported by the Regione Campania NEURORARE project (DGR 393 del 19/07/2022).

## CONFLICT OF INTEREST STATEMENT

None of the authors has any conflict of interest to disclose. We confirm that we have read the Journal's position on issues involved in ethical publication and affirm that this report is consistent with those guidelines.

## Supporting information


Table S1.

## Data Availability

The data that support the findings of this study are available on request from the corresponding author. The data are not publicly available due to privacy or ethical restrictions.
