# Supplementary material for: Potassium current inactivation as a novel pathomechanism for KCNQ2 developmental and epileptic encephalopathy
Source: Epilepsia. 2025 Apr 28;66(6):e98–e105. doi: 10.1111/epi.18427 (PMC12169393; doi:10.1111/epi.18427)
Supplement: Supplementary file 1 — Table S1. [file EPI-66-e98-s001.pptx]

## Slide 1
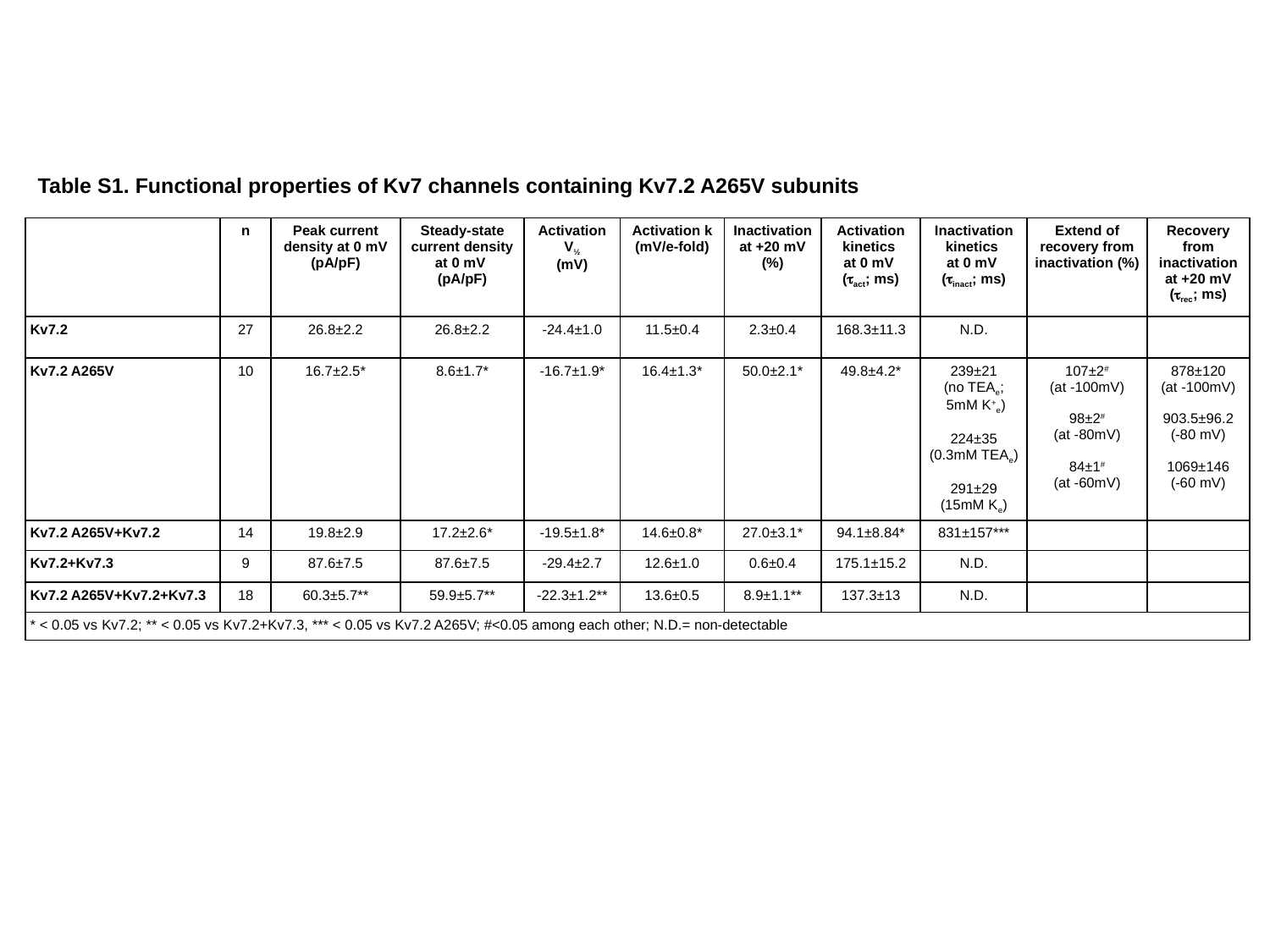

Table S1. Functional properties of Kv7 channels containing Kv7.2 A265V subunits
| | n | Peak current density at 0 mV (pA/pF) | Steady-state current density at 0 mV (pA/pF) | Activation V½ (mV) | Activation k (mV/e-fold) | Inactivation at +20 mV (%) | Activation kinetics at 0 mV (tact; ms) | Inactivation kinetics at 0 mV (tinact; ms) | Extend of recovery from inactivation (%) | Recovery from inactivation at +20 mV (trec; ms) |
| --- | --- | --- | --- | --- | --- | --- | --- | --- | --- | --- |
| Kv7.2 | 27 | 26.8±2.2 | 26.8±2.2 | -24.4±1.0 | 11.5±0.4 | 2.3±0.4 | 168.3±11.3 | N.D. | | |
| Kv7.2 A265V | 10 | 16.7±2.5\* | 8.6±1.7\* | -16.7±1.9\* | 16.4±1.3\* | 50.0±2.1\* | 49.8±4.2\* | 239±21 (no TEAe; 5mM K+e)   224±35 (0.3mM TEAe)   291±29 (15mM Ke) | 107±2# (at -100mV)   98±2# (at -80mV)   84±1# (at -60mV) | 878±120 (at -100mV)   903.5±96.2 (-80 mV)   1069±146 (-60 mV) |
| Kv7.2 A265V+Kv7.2 | 14 | 19.8±2.9 | 17.2±2.6\* | -19.5±1.8\* | 14.6±0.8\* | 27.0±3.1\* | 94.1±8.84\* | 831±157\*\*\* | | |
| Kv7.2+Kv7.3 | 9 | 87.6±7.5 | 87.6±7.5 | -29.4±2.7 | 12.6±1.0 | 0.6±0.4 | 175.1±15.2 | N.D. | | |
| Kv7.2 A265V+Kv7.2+Kv7.3 | 18 | 60.3±5.7\*\* | 59.9±5.7\*\* | -22.3±1.2\*\* | 13.6±0.5 | 8.9±1.1\*\* | 137.3±13 | N.D. | | |
| \* < 0.05 vs Kv7.2; \*\* < 0.05 vs Kv7.2+Kv7.3, \*\*\* < 0.05 vs Kv7.2 A265V; #<0.05 among each other; N.D.= non-detectable | | | | | | | | | | |

## Slide 2
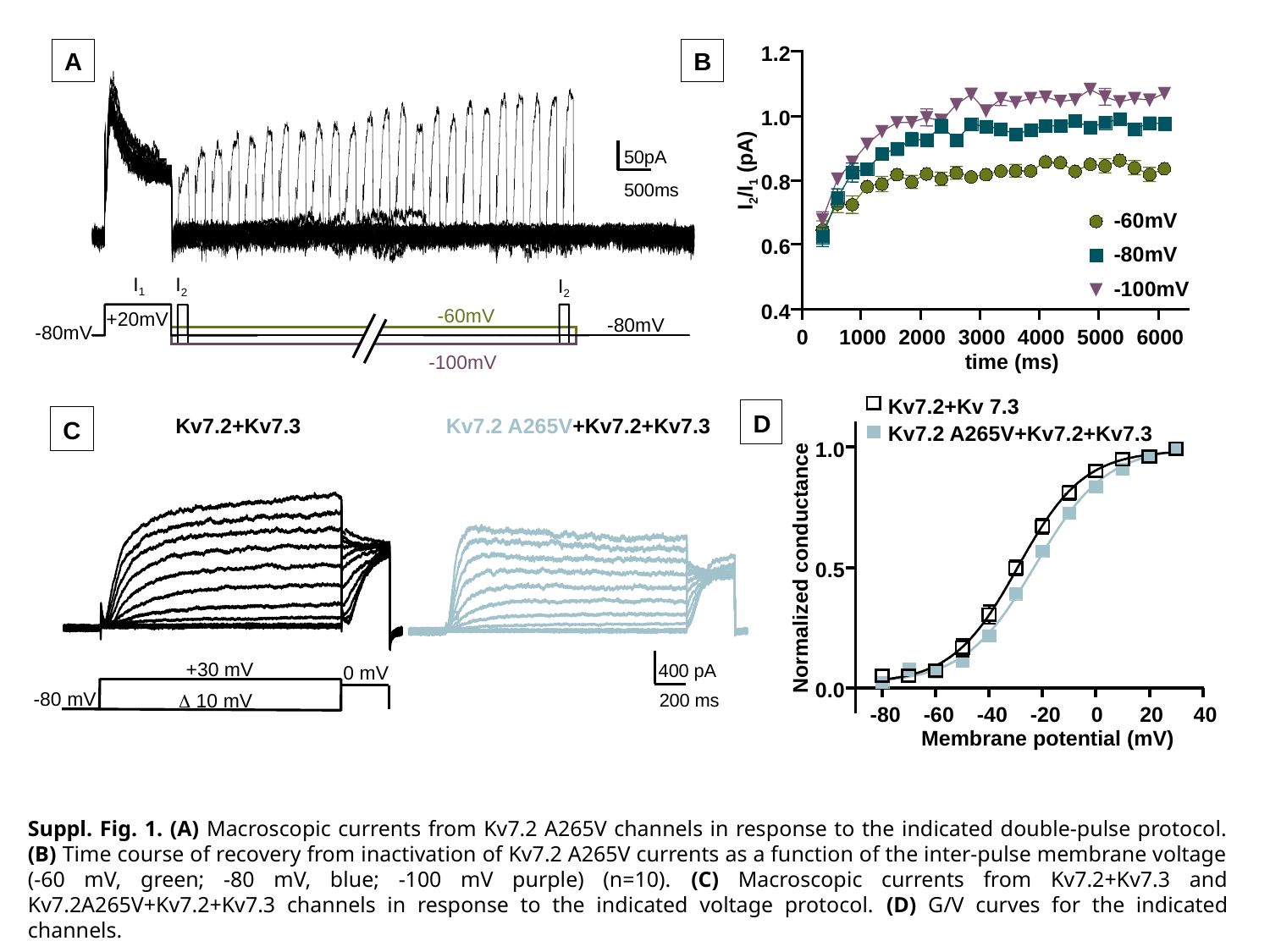

1.2
1.0
0.8
0.6
0.4
0
1000
2000
3000
4000
5000
6000
I2/I1 (pA)
-60mV
-80mV
-100mV
time (ms)
A
B
v
v
50pA
500ms
I1
I2
I2
-60mV
+20mV
-80mV
-80mV
-100mV
Kv7.2+Kv 7.3
Kv7.2 A265V+Kv7.2+Kv7.3
1.0
0.5
Normalized conductance
0.0
-80
-60
-40
-20
0
20
40
Membrane potential (mV)
D
Kv7.2+Kv7.3
Kv7.2 A265V+Kv7.2+Kv7.3
C
200 ms
+30 mV
0 mV
-80 mV
D 10 mV
400 pA
Suppl. Fig. 1. (A) Macroscopic currents from Kv7.2 A265V channels in response to the indicated double-pulse protocol. (B) Time course of recovery from inactivation of Kv7.2 A265V currents as a function of the inter-pulse membrane voltage (-60 mV, green; -80 mV, blue; -100 mV purple) (n=10). (C) Macroscopic currents from Kv7.2+Kv7.3 and Kv7.2A265V+Kv7.2+Kv7.3 channels in response to the indicated voltage protocol. (D) G/V curves for the indicated channels.
